# The McMaster Toronto Arthritis patient preference questionnaire (MACTAR): a methodological study of reliability and minimal detectable change after a 6 week-period of acupuncture treatment in patients with rheumatoid arthritis

**DOI:** 10.1186/s13104-017-2991-0

**Published:** 2017-12-04

**Authors:** Nina Brodin, Wilhelmus J. A. Grooten, Sara Stråt, Elin Löfberg, Helene Alexanderson

**Affiliations:** 1 Division of Physiotherapy, Department of Neurobiology, Care Sciences and Society, Karolinska Institutet, Huddinge, Sweden; 2 Division of Physiotherapy, Department of Orthopaedics, Danderyd Hospital, Stockholm, Sweden; 3 Functional Area Occupational Therapy and Physical Therapy, Karolinska University Hospital, Solna, D2:01, 171 76 Stockholm, Sweden

**Keywords:** Interview, Patient preference, Rehabilitation, Responsiveness, SSED

## Abstract

**Objectives:**

The McMaster Toronto Arthritis patient preference questionnaire (MACTAR) is a semi-structured interview consisting of a baseline and a follow-up interview. The MACTAR baseline is reliable and valid, however the reliability of the MACTAR follow-up is scarcely described. The aim of this study was to describe aspects of reliability and ability to detect changes of the Swedish MACTAR follow-up following acupuncture treatment in individuals with rheumatoid arthritis.

**Results:**

The study was of Single Subject Experimental Design, with a 2-week non-interventional A-phase and a 6-week intervention B-phase. Eight individuals with RA, age 30–68 years, were included. MACTAR baseline was performed once followed by five assessments with MACTAR follow-up during the A-phase and another ten assessments during the B-phase. Reliability statistics were calculated for measurements 1–3 during the A-phase and the ability to detect effects of acupuncture treatment was tested by celeration lines in the B-phase. The MACTAR follow-up was highly reliable (ICC = 0.7–0.9, SEM = 2.3–4.3, and SDD = 6.2–11.7). Visual and statistical analyses indicated that the MACTAR follow-up could detect effects on individual- and group levels after acupuncture treatment, indicating that the MACTAR follow-up seems to be reliable and is able to detect effects of acupuncture treatment in RA.

**Electronic supplementary material:**

The online version of this article (10.1186/s13104-017-2991-0) contains supplementary material, which is available to authorized users.

## Introduction

Rheumatoid arthritis (RA) is an inflammatory rheumatic condition with polyarthritis leading to pain, swollen and stiff joints, fatigue, and disability [1–3]. Acupuncture might reduce pain and reduce inflammation in patients with RA [4].

Reliable and valid clinical outcome measures are a prerequisite for assessment of outcome and effects of treatments. Several patient-reported outcome measures (PROMs) are developed and/or validated for patients with RA, but very few focus on patient preference [5]. PROMs with pre-defined questions/items might not be relevant for all individuals with RA [6–8]. A patient preference instrument could be more sensitive to detect changes than recommended PROMs [6, 9].

The objective of this study was to establish the reliability of the Swedish McMaster Toronto Arthritis patient preference questionnaire (MACTAR) follow-up interview and to describe its ability to detect changes after a 6 week-period of acupuncture treatment in patients with RA.

## Main text

### Background

The MACTAR was the first patient preference instrument developed for patients with RA [10]. It was revised into a semi-structured baseline and follow-up interview in the Netherlands [9], and is sensitive to change following both medical treatment and exercise in RA [6, 11] and in chronic low back pain [12]. The MACTAR is a valid measure for myositis and RA [13, 14] and for hip- and knee osteoarthritis [15]. However, the MACTAR follow-up has not previously been evaluated for all aspects of reliability, as to sensitivity to change or to ability of detecting changes following acupuncture treatment in RA.

### Study design

This is a single subject experimental design (SSED) study which in contrast to an open label design with group analysis allows each patient to be their own control by including a non-interventional A-phase followed by an interventional B-phase [16]. Patients were assessed systematically three times a week during the 2-week A-phase and twice a week during the 6-week B-phase.

### Patients

All patients with RA, referred to acupuncture treatment for pain, at Danderyd Hospital, Stockholm (n = 10), during August 2006 to January 2007, who fulfilled the inclusion criteria were eligible and were invited to participate. Inclusion criteria; RA diagnosis according to the ACR criteria [17], diagnosis duration > 12 months, ≤ 70 years of age, unchanged medication during the past 3 months. Exclusion criteria; any contra-indication for acupuncture treatment; received acupuncture treatment during the past 6 months, not understanding the Swedish language. All 10 patients accepted participation initially, however, two patients chose to abort participation, due to lack of time or starting a new medical treatment at the time of inclusion. Eight patients entered and completed the study and their demographic data is presented in Tables 1 and 2.

**Table 1:** Demographic data of the eight participants with RA and individual pre- and post-acupuncture self-reported assessments

ID	Gender	Age, years	RA duration, years	Living situation	Work/sick leave	Medication	MACTAR median 1 and 2, A-phase		MACTAR		VAS pain, 0–100		VAS, 0–100 Well-being	
							A1	A2	B1	B10	B1	B10	B1	B10
1	F	68	2	Living with partner	Retired	Cox^a^, Dmard^b^	45	43	43	41	69	75	76	70
2	M	56	1.5	Living with partner	Work 20%	Dmard^b^	54	54	54	60	43	49	43	42
3	F	63	10	Living alone	Sick-leave 100%	Dmard^b^, TNF^c^, kortison^d^	52	52	55	65	29	10	30	15
4	F	57	2.5	Living with partner	Work 100%	Dmard^b^	55	56	56	47	38	57	17	60
5	F	54	6	Living alone	Work 50%	Dmard^b^	40	52	56	62	49	50	34	51
6	M	65	24	Living with partner	Work 75%	Cox^a^, Dmard^b^	54	55	55	63	18	9	17	10
7	M	63	30	Living with partner	Sick-leave 100%	Cox^a^, TNF^c^	54	52	50	56	8	10	16	11
8	F	30	20	Living with partner	Work 50%	Cox^a^	44	43	44	65	62	42	71	35

*B1* first assessment during the B-phase, *B10* last assessment in the B-phase after 10 acupuncture treatments

^a^Cyclooxygenase inhibitor (COX-inhibitor)

^2^Disease modifying anti rheumatic drugs (DMARD)

^3^Tumor necrosis factor (TNF-inhibitor)

^4^Glucocorticoids (prednisolone)

**Table 2:** MACTAR and other assessments at different time-points during the A- and B-phase

Measure	A1 Md (Q1–Q3) n = 8	B1 Md (Q1–Q3) n = 8	B10 Md (Q1–Q3) n = 8	p value B10 vs B1
MACTAR, 21–77	53.0 (43.0–54.0)	54.5 (47.0–55.5)	61.0 (51.5–64.0)	p = 0.02
VAS, 0–100				
Pain	Na	40.5 (23.5–55.5)	45.5 (10.0–53.5)	NS
Well-being	Na	32.0 (17.0–54.0)	38.5 (13.0–55.5)	NS

*A1* first assessment in the A-phase, *B1* first assessment in the B-phase, *B10* 10th assessment in the B-phase after completed acupuncture treatment, *MACTAR* McMaster Toronto Arthritis, *VAS* Visual Analogue Scale, *na* not assessed

### Assessments

The MACTAR is a semi-structured interview assessing activity limitation, consisting of a baseline interview and a follow-up interview. Both interviews contain pre-defined questions on general health, physical function, social function and emotional function which are rated according to degree of disease-impact in daily life on a five-grade Likert Scale from 1 (poor health) to 5 (good health) [9, 13]. Patients are also asked to state five activities of daily living that are limited due to RA, and then to rank the five activities starting with the most important to improve. In the follow-up interview, patients rate if their ability to perform their five activities has improved, deteriorated or not changed at all. Patients also rate if their general health, physical-, social-, or emotional function has changed due to the treatment. MACTAR total score varies from 21 (severe limitations) to 77 (no limitation).

Pain during the last week was assessed on a Visual Analogue Scale (VAS pain), 0 (no pain) − 100 (worst imaginable pain) [18]. Patients’ global well-being during the last week (PGA) was rated on a VAS, 0 (best well-being possible) − 100 (poor well-being) [19].

### Procedures

At the initial visit, patients were assessed using the MACTAR baseline interview which took between 20 and 45 min to complete. Five telephone interviews were then scheduled during the following 2 weeks during the A-phase. The MACTAR follow-upwas used during these telephone interviews, and then throughout the rest of the study. The participants were encouraged to set aside 15 min in private during the telephone interviews. After completing these initial six interviews (A-phase with one baseline and five follow-up interviews) the acupuncture treatment was introduced twice a week during the first 4 weeks, and then once a week during the following 2 weeks (B-phase). Each patient received 10 acupuncture treatments. The MACTAR follow-up interview was longitudinally compared to the VAS pain and to PGA. Thus the MACTAR follow-up interview, VAS pain and PGA were performed at every treatment visit. One physical therapist performed all acupuncture treatments, and another physical therapist administered all assessments. Both physical therapists had vast experience of acupuncture treatment and of using the included assessment methods.

### Data analysis

Due to the type of data, non-parametric statistics were used in all statistical analyzes and data on group level are presented as median and range. Intra Class Correlation Coefficients were calculated between the first three measurements during the A-phase (A1 vs A2, A1 vs A3, and A2 vs A3), as well as the standard error of the measurement (SEM), the coefficient of variation expressed as percentage of the mean (CV%), and the smallest detectible difference (SDD). Bland and Altman methods were used to assess possible systematic disagreement between the test occasions [20]. Calculations included the mean difference between the measures, the standard deviation of the differences (SD difference) and the 95% limits of agreement: mean ± 2 SD difference. Intra Class Correlation coefficients of ICC > 0.75 were considered to reflect “good” to “excellent” correlations [21]. To assess sensitivity to change, two different procedures were undertaken. Firstly, to analyze changes in the MACTAR follow-up interview during the B-phase compared to the A-phase, two median values (one median of the first three assessments and one median of the remaining three assessments) from the six A-phase assessments were calculated, and a celeration line was drawn through these median values continuing through the 10 B-phase assessments. A majority of assessment points during the B-phase above or below the celeration line indicate a change in activity limitation [16]. A classic power analysis based on mean values and sample size are not applicable for a SSED. Instead, the number of measurement points in both A- and B-phases and the natural variation during the A-phase indicates how many patients need to be included. A large A-phase measurement point variation requires a large change during the B-phase to indicate a true change. In SSED design results can be calculated for one patient, but the replication of results in a small number of additional patients is essential in SSED [22, 23]. Secondly, the Friedman’s ANOVA test was performed to analyze changes during the B-phase on the MACTAR follow-up interview, VAS pain and PGA, with the Wilcoxon signed rank test as after test. The level of significance was set to p < 0.05. SPSS for Windows, version 22, was used in all analyses. Statsoft, Statistica (version 12) was used to create the Bland and Altman plots.

### Results

All eight participants completed the 10 acupuncture treatments and all assessments throughout both the A-phase and the B-phase.

The ICC between A1 and A2 was 0.747, with SEM 4.21, CV% 8.60 and SDD 11.68. For the measures A1–A3, ICC was 0.697 with SEM 4.16, CV% 8.50 and SDD 11.50 and for the A2–A3 the ICC was 0.878 with SEM 2.25, CV% 4.50 and SDD 6.24, indicating good to excellent reliability.

**Fig. 1:**
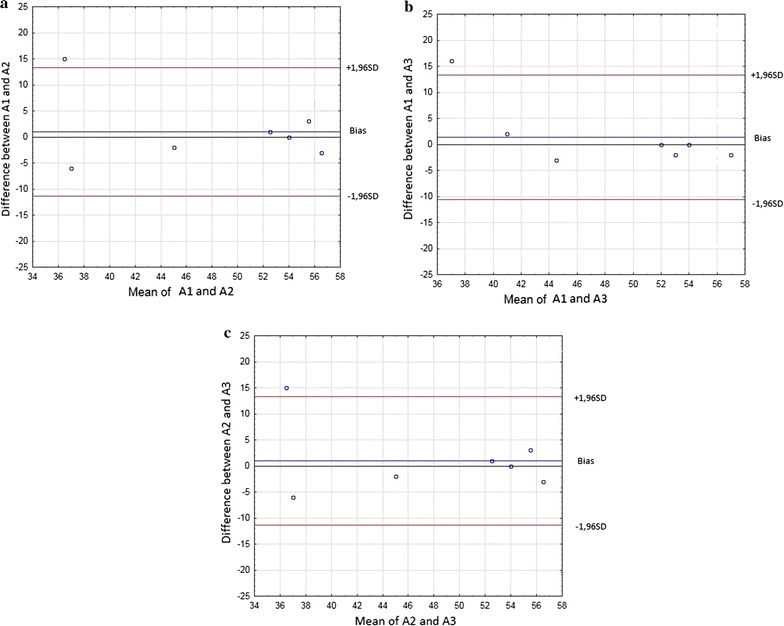
**a** Bland–Altman plot assessing possible systematic disagreement between the two test occasions A1 and A2. **b** Bland–Altman plot assessing possible systematic disagreement between the two test occasions A1 and A3. **c** Bland–Altman plot assessing possible systematic disagreement between the two test occasions A2 and A3

Figure 1a–c with the Bland–Altman plots shows the difference between the occasions plotted against the mean of the measurement points A1–A2, A1–A3, and A2–A3. There was no systematic disagreement between the test occasions.

The celeration line analyses indicated that the MACTAR follow-up interview could detect changes after treatment, as all participants except one had a majority of assessment points above or below the celeration line in the B-phase (Additional file 1: Figure S1). Analysis on group level revealed a statistically significant improvement in the MACTAR follow-up interview at B10 compared to B1 (p = 0.02), while VAS pain and PGA remained unchanged (Table 2).

### Discussion

The present study indicated good to excellent reliability and ability to detect changes over time for the MACTAR follow-up without systematic disagreement between the test occasions. Best reliability, i.e., high ICC, low SEM, CV% and low SDD were found for measurements A2 and A3, which implies that it is preferable to exclude the first session of the MACTAR follow-up interview in clinical daily routines. The MACTAR follow-up interview was able to detect effect of acupuncture treatment, while measures of pain and well-being remained unchanged suggesting that the patient-preference MACTAR is a valuable addition to predefined PROMs.

The celeration line analysis indicated a change in MACTAR score in seven participants, which supports the statistically significant change on group level. These changes were not mirrored in the VAS pain or PGA, which could indicate that the MACTAR follow-up interview was more sensitive to change as it captures values that are important to the patient beyond those assessed using general PROM’s. The MACTAR follow-up interview was highly responsive following both medical treatment and exercise in patients with RA [6, 9]. Significant within-group improvement in MACTAR follow-up was evident also in an exercise study in patients with myositis [24].

### Methodological considerations

Activities once identified as important to improve by using the MACTAR, might lose relevance as seasons and other life factors change [6, 12, 13]. Although during a short time-span, our study was performed during fall and winter when patients with RA often experience day-to-day variations due to for example infections or weather changes [25]. In some patients, this led to large variations in the A-phase assessment of patient preference, VAS pain and PGA. Assessments during warmer seasons might have resulted in smaller symptom variation further improving reliability and sensitivity to change of the MACTAR follow-up. A SSED design might not be optimal in RA-patients, however a similar study protocol was successfully performed in patients with other inflammatory conditions [26, 27].

The first MACTAR interview was performed during the first study visit, while the following five A-phase interviews were performed over the telephone. One advantage was that telephone interviews required no time for traveling to and from the clinic, which probably enabled a more diverse group of full-time workers and severely disabled patients to participate. Our study included both younger and older men and women with various RA-duration, which strengthened external validity of our results. However, non-verbal communications are lost and participants might have had difficulties to find a secluded space to avoid distractions during the interviews. However, a pilot telephone interview performed with one participant before the first study visit did not reveal any test–retest variations. Although RA is not a rare condition, a SSED design was chosen to be able to study the natural variability of patient preference on an individual and on a group level. In order to account for this natural variability, a relative large number of subjects were included for this SSED compared to common study sizes in SSED [22]. The SSED has a relative low evidence value and a larger study with another design is therefore needed to confirm our results. In the present study, patients were treated with manual or electrical acupuncture based on clinical status and indication. The choice of stimulation module might not be important as the main purpose of this study was to evaluate measurement properties of the MACTAR.

In conclusion, the MACTAR follow-up interview seems to be reliable and may be able to detect changes in activity limitations following acupuncture treatment in patients with established RA. This implies that the MACTAR could be a valuable addition to already established outcome measures. The patient-preference focus will enhance patient relevance and patients’ participation in clinical care. For optimal precision, we suggest a learning occasion before the first MACTAR follow-up assessment.

## Limitations

The main limitations of this study are the smaller sample-size SSED and that assessments were only performed during the colder season with more day-to-day symptom variation which might have resulted in lower validity and sensitivity to change of the MACTAR.

## Additional file

**Additional file 1: Figure S1.** Individual MACTAR data on all 8 participants. A celeration line is drawn through the two median values during the six A-phase assessments. The celeration line is then extended through the 10 B-phase assessments. A statistically significant change is defined as 9/10 assessments in the B-phase over or under the celeration line. BL = baseline interview, A1–A5 = assessments 1–5 during the A-phase, B1–B10 = assessments during the interventional B-Phase.

## Abbreviations

MACTAR: McMaster Toronto Arthritis patient preference questionnaire
RA: rheumatoid arthritis
HAQ: Health Assessment Questionnaire
AIMS: Arthritis Impact Measurement Scale
PROM: patient-reported outcome measure
SSED: single subject experimental design
VAS: Visual Analogue Scale
PGA: patient’s global well-being
SEM: standard error of the measurement
CV%: the coefficient of variation expressed as percentage of the mean
SDD: smallest detectible difference
SD: standard deviation
ICC: intra-class correlation
